# Dehumanization During the COVID-19 Pandemic

**DOI:** 10.3389/fpsyg.2021.634543

**Published:** 2021-02-11

**Authors:** David M. Markowitz, Brittany Shoots-Reinhard, Ellen Peters, Michael C. Silverstein, Raleigh Goodwin, Pär Bjälkebring

**Affiliations:** ^1^School of Journalism and Communication, University of Oregon, Eugene, OR, United States; ^2^Center for Science Communication Research, University of Oregon, Eugene, OR, United States; ^3^Department of Psychology, The Ohio State University, Columbus, OH, United States; ^4^Department of Psychology, University of Oregon, Eugene, OR, United States; ^5^Department of Psychology, University of Gothenburg, Gothenburg, Sweden

**Keywords:** dehumanization, COVID-19, pandemic, risk perceptions, conspiracy beliefs

## Abstract

Communities often unite during a crisis, though some cope by ascribing blame or stigmas to those who might be linked to distressing life events. In a preregistered two-wave survey, we evaluated the dehumanization of Asians and Asian Americans during the COVID-19 pandemic. Our first wave (March 26–April 2, 2020; *N* = 917) revealed dehumanization was prevalent, between 6.1% and 39% of our sample depending on measurement. Compared to non-dehumanizers, people who dehumanized also perceived the virus as less risky to human health and caused less severe consequences for infected people. They were more likely to be ideologically Conservative and believe in conspiracy theories about the virus. We largely replicated the results 1 month later in our second wave (May 6–May 13, 2020; *N* = 723). Together, many Americans dehumanize Asians and Asian Americans during the COVID-19 pandemic with related perceptions that the virus is less problematic. Implications and applications for dehumanization theory are discussed.

## Dehumanization During the COVID-19 Pandemic

Communities often unite during a crisis (Berke et al., 1993; Tan and Enderwick, 2006), though one coping strategy is to blame or stigmatize others linked to a crisis (Lau et al., 2020). History suggests scapegoating is quite common; for example, Muslim Americans were blamed for the terrorist attacks of September 11th, 2001 (Peek, 2010), and the African lifestyle was blamed for Ebola in the 1990s (Joffe and Haarhoff, 2002). Scapegoating can offer several benefits to individuals. It can serve as a way to emotionally regulate negative life events (Garnefski et al., 2001), a way to avoid threat by blaming others (Tennen and Affleck, 1990), and as a mechanism to ascribe blame to reduce uncertainty (Petersson, 2003). Scapegoating can also introduce societal costs. During the COVID-19 pandemic, Asians and Asian Americans have become the victims of physical and verbal attacks (Hong, 2020; Tavernise and Oppel, 2020; Zheng, 2020), even from United States government officials (Somvichian-Clausen, 2020), because of their ethnicity and the virus’ origins. Indeed, the humanity of Asians and Asian Americans is being violated, denied, or questioned during this pandemic, not unlike other periods of history when hated outgroups were perceived as “less than human” (Musolff, 2014, 2015; Slovic and Lin, 2018). To mitigate hatred and cruelty around the world, it is important to identify blind spots and realize how we treat others during global crises.

As the COVID-19 pandemic progresses, it is particularly crucial to document whether people inappropriately blame certain groups for the pandemic and treat them unfairly and cruelly as a result. It is also crucial to understand who perpetuates this blame. In a large-scale, preregistered, two-wave study, we evaluated the blatant dehumanization of Asians and Asian Americans during the pandemic. We found that many people dehumanized Asians and Asian Americans. People who dehumanized tended to have lower risk perceptions toward the virus, were more ideologically conservative, and believed in conspiracy theories and disinformation about the virus compared to non-dehumanizers.

### A Primer on Dehumanization Research

Dehumanization, or the blatant denial of a group’s humanity or humanness, is a global issue perpetrated on a range of outgroups (Haslam, 2006; Haslam and Loughnan, 2014). For example, Americans tend to rate Muslims as less evolved compared to other groups such as Arabs or Australians (Kteily et al., 2015), and perceive immigrants as less than fully evolved humans who deserve punishment for their actions (Markowitz and Slovic, 2020b). Dehumanization can be tacit through people denying an outgroup’s secondary emotions (e.g., emotions considered unique to humans such as nostalgia; Leyens et al., 2003; Haque and Waytz, 2012), or explicit through callous metaphors that describe an outgroup (e.g., calling immigrants animals; see Markowitz and Slovic, 2020b). Dehumanization often goes beyond hatred of an outgroup, however (Bruneau et al., 2019). It can reflect one group believing outgroup members deserve to suffer or receive harsh treatment (e.g., as with some Americans towards immigrants; Fiske and Rai, 2014) or are less worthy of rights and treatment ascribed to other humans.

#### Antecedents of Dehumanization

People who dehumanize come from a variety of social, psychological, and demographic paths (Haslam and Loughnan, 2014; Markowitz and Slovic, 2020b). People who blatantly dehumanize immigrants, for example, tend to feel dominant over them (Pratto et al., 1994), are older (Markowitz and Slovic, 2020b) and more ideologically conservative (Utych, 2018). That is, compared to non-dehumanizers, people who dehumanize are more likely to support right-leaning policies in the United States, such as immigration raids and the death penalty for convicted murderers (Markowitz and Slovic, 2020b). Several characteristics are antecedents of dehumanization, suggesting similar indicators might help to identify those who dehumanize Asians and Asian Americans during the COVID-19 pandemic.

#### Consequences of Dehumanization

People who dehumanize are often less prosocial toward perceived outgroups (Haslam and Loughnan, 2014). For example, Cuddy et al. (2007) revealed that those who denied secondary emotions (e.g., anguish, remorse) to Hurricane Katrina victims reported they would be less likely to volunteer time to help needy individuals. Other work finds people who perceived Muslims as animals were less likely to support reparation policies for them (Zebel et al., 2008). In general, people who dehumanize attend to outgroups less and also support them less compared to non-dehumanizers (Dickert and Slovic, 2009; Harris and Fiske, 2011).

People who dehumanize also tend to favor cruelty and ascribe harsher punishments toward outgroups. Markowitz and Slovic (2020b) observed that people who dehumanized immigrants would sentence them to more jail time if they were caught crossing the United States-Mexico southern border. Other research finds that people who dehumanize tend to ascribe criminals with more blame for their actions than non-dehumanizers (Bastian et al., 2013). Finally, those who dehumanize also display less empathy toward outgroups (e.g., homeless people; Herrera et al., 2018) and generally report experiencing heightened negative affect or disgust toward them (Haslam, 2006; Buckels and Trapnell, 2013; Bruneau et al., 2018).

Together, people who dehumanize often believe that an outgroup is less deserving of rights and feelings than an ingroup. Dehumanization is linked to a range of social, psychological, and demographic antecedents, with far-reaching social and psychological consequences.

### Summary of Our Research Aims

Our research draws on the prior perspectives to achieve two goals. First, we attempt to measure the dehumanization of Asians and Asian Americans during early stages of the COVID-19 pandemic in an online sample of Americans. Hatred toward Asians during the pandemic is widespread in the United States, but blatant dehumanization is a concept that considers the overt denial of another group’s humanness. This different psychological concept should be empirically documented, and we address this opportunity in the current investigation.

**FIGURE 1:**
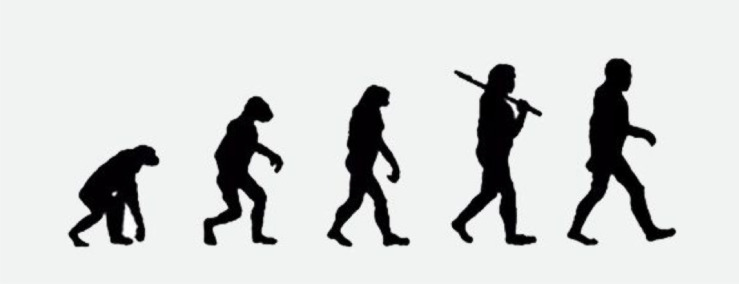
The Ascent of Man scale.

Importantly, prior work also suggests outside of this or any pandemic, some people dehumanize Asians (e.g., Chinese people, South Koreans) compared to Americans. A landmark paper by Kteily et al. (2015; Study 1) had participants judge the humanness of many outgroups and they rated Chinese people (Cohen’s *d* = 0.18) and South Koreans (Cohen’s *d* = 0.23), but not Japanese people (Cohen’s *d* = 0.02), as less than human on the Ascent of Man (AOM) scale relative to Americans. The AOM contains five hominids that become more evolved and human-like by walking upright and having straighter legs (see Figure 1). Therefore, the average American might perceive Asians as less evolved than Americans (e.g., Slovic and Lin, 2018); we seek to identify the rate of dehumanization during this critical period (e.g., the percent of people in our samples who dehumanized) and who tends to dehumanize during a pandemic.

A second goal is to recruit risk perceptions associated with the virus and how people felt during the COVID-19 pandemic, linking such variables to the dehumanization of Asians and Asian Americans. Risk perceptions are often used to understand how people make decisions under conditions of uncertainty (e.g., about their health or money), but they are less often connected to how people think about target groups or their humanness (though, see Schaller et al., 2003; Faulkner et al., 2004 for notable exceptions). We attempt to understand how personal risk assessments, specifically risk perceptions about COVID-19, might associate with how people treat groups who are stigmatized and blamed for COVID-19 because of its origins. If we can understand the people who dehumanize, how they think about the virus, and its associated risk, we can potentially mitigate undeserving cruelty toward purported outgroups.

This work is important for dehumanization theory and has many practical implications as well. First, dehumanization research often measures rates of blatant dehumanization cross-sectionally. Multi-wave evaluations are rare (but see Kteily et al., 2015; Pizzirani and Karantzas, 2019 for examples), and we measure the time-dependent nature of dehumanization and its effects during a global crisis. Second, it is unclear if dehumanization might be amplified during periods of heightened unrest and uncertainty. Risk perception models suggest that this idea is plausible. The social amplification of risk framework argues that crises or hazards are shaped by social, psychological, and cultural factors relevent to a particular event (Kasperson et al., 1988; Pidgeon et al., 2003). Social amplifiers (e.g., scientists, the media) can increase or decrease perceived risks, uncertainties, or emotional responses associated with a harmful event (p. 181). For example, people might read about the virus’ origins in China, increase their negative perceptions of Asians and Asian Americans, and perceive them as less-than-human during this tense period in history; thus, they might feel more threatened by COVID-19, at least in the short-term. We test how Asians and Asian Americans are dehumanized in a time when risk and uncertainty is elevated.

Finally, it is practically important to identify those who treat others as less than human during a global crisis. Such evaluations might provide warning signs about people who dehumanize and aid in interventions to encourage humane treatment of perceived outgroups.

### Predictions

We use prior theory and evidence to predict relations between dehumanization, perceptions of risk, and analyses of unrestrained descriptions of people’s thoughts and feelings about the virus. All hypotheses were preregistered on the Open Science Framework^1^. Our preregistrations for other associated papers are located at the same link. Note, the data did not support most predictions that follow, though we explain our original logic, present the results, and wrestle with the differences between predictions and results.

We expect that perceived risk and COVID-19 severity will be positively related to dehumanization of Asians and Asian Americans based on prior work that suggests people may dehumanize others to cope with repeated social and psychological distress (Schulman-Green, 2003; Haque and Waytz, 2012). Those who believe COVID-19 is more of a problem might therefore look for blameworthy targets and show strong dehumanization tendencies toward them (see Buckels and Trapnell, 2013). We hypothesized that:

H_1_: Higher rates of blatant dehumanization are associated with more perceived risk, severity, and negative emotion toward COVID-19.

People who dehumanize immigrants tend to write about them and other immigration issues in more impersonal terms (Markowitz and Slovic, 2020b). Presumably, people who dehumanized also wanted to increase the social and psychological distance between them and immigrants; this desire can be achieved or reflected in an increased rate of impersonal pronouns (e.g., *it*, *who*, *anyone*) in a person’s speech (see Pennebaker, 2011). Therefore, consistent with other evidence, we predicted:

H_2_: Blatant dehumanization is associated with an increase in impersonal pronouns.

We further expected that those who display generally high levels of intrinsic motivation during the COVID-19 pandemic will also dehumanize less than those who display generally low levels of intrinsic motivation. According to self-determination theory (Deci and Ryan, 1985), people tend to act in accordance with goals that are internalized (or self-determined) compared to goals that are not (Deci and Ryan, 2002). Self-determination theory argues for a continuum of self-determination to direct behavior, where goals that are intrinsically motivated (e.g., the most self-determined) tend to be satisfying and easier to pursue than those that are forced or “amotivating” (e.g., the least self-determined).

Self-determination has important connections to how people treat and perceive target outgroups. For example, people who are self-determined or more intrinsically motivated to regulate their prejudice toward Black people tend to demonstrate lower levels of implicit racial bias (e.g., via the Implicit Association Test) and explicit prejudice (e.g., via the Symbolic Racism 2000 Scale) than those who are less self-determined to regulate their prejudice (Legault et al., 2007). Other complementary work suggests that high self-determination and motivation is linked to prosocial behavior. For example, sustained prosociality (e.g., volunteering) often occurs when intrinsic motivation are high compared to low (Aydinli et al., 2016), and people who are intrinsically motivated to control their own prejudice toward others can overcome the activation of racial biases even when cognitively depleted (Park et al., 2008). Self-determination and motivation are therefore crucial to indicate how people feel or behave toward others, particularly those who might be the target of prejudice, racism, or dehumanization.

Consistent with prior evidence supported by self-determination theory, we expect that people who write with more verbal traces of self-determination during the COVID-19 pandemic will dehumanize target outgroups less. We measure self-determination and motivation through writing style patterns, particularly a dimension called *verbal drives*, indicated by terms such as *accomplish*, *master*, and *success*, which has been validated in prior research (Schultheiss, 2013). We predict *verbal drives* will be associated with less dehumanization toward Asians and Asian Americans. While we cannot precisely identify the type of intrinsic motivation people are experiencing or what people might be specifically motivated about during this time, our hypothesis is consistent with the idea that high levels of implicit motivation are associated with less prejudice and hatred toward an outgroup, and therefore, we expect less dehumanization.

H_3_: Less dehumanization is associated with more verbal drives.

We also explored how conspiracy beliefs and one’s objective numeric ability link to dehumanization, and how effects might change over time. Prior work suggests highly numerate individuals are more logical decision makers in hypothetical scenarios (Peters et al., 2006) and in medical decision making (Reyna et al., 2009). We explored how objective numeracy, or the idea that people are adept in dealing with numbers and mathematical operations (Peters and Bjälkebring, 2015), relates to dehumanization. It is plausible that those higher in objective numeracy are more logical thinkers (vs. emotional thinkers) during a time of uncertainty and therefore dehumanize less than those who are lower in objective numeracy.

Taken together, in this work we evaluate rates of dehumanization during the COVID-19 pandemic toward Asians and Asian Americans, understand the relationship between dehumanization and risk perceptions during the early stages of the pandemic, and profile those who dehumanize Asians and Asian Americans. One way to reduce global hatred is to identify those who might treat others as less-than-human and assess dehumanization prevalence. We take up this opportunity in the current investigation.

## Method

**FIGURE 2:**
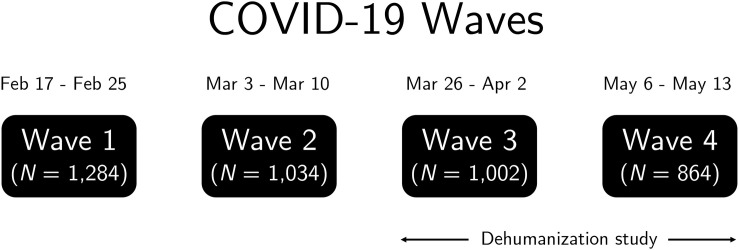
UO-EPIDeMIC Study (Emotions & Polarization In Decisions & Media In COVID-19) timeline across waves. Sample sizes reflect the total number of participants recruited, though final samples are reported in the main text.

We collected two waves of survey data to evaluate how dehumanization operates cross-sectionally and at different time-points. This project is part of the UO-EPIDeMIC Study (Emotions & Polarization In Decisions & Media In COVID-19), a series of investigations with the same survey instrument that collected information about people’s thoughts, feelings, and reactions to the COVID-19 pandemic over time. This is our only study to use the AOM items. Figure 2 displays survey waves and those relevant to the current study (Wave 3 is henceforth labeled as AOM Time 1 and Wave 4 is AOM Time 2).

AOM Time 1 ran from March 26, 2020 until April 2, 2020 and started with 1,002 participants. Consistent with best practices for analyzing language data, we excluded participants whose writing samples contained ≤15 words (*n* = 85) because small word counts can inflate the prevalence of word categories. Our final AOM Time 1 sample contained 917 participants. AOM Time 2 ran from May 6, 2020 to May 13, 2020 and started with 864 participants in the survey, though only some participants completed AOM Time 1 (final *n* = 723 after exclusions).

Participants were recruited from CloudResearch, an extension of Amazon Mechanical Turk, and paid at least $3.00 for their participation in both 25- to 30-min sessions. In AOM Time 1, we started with 433 self-identified males and 474 self-identified females (some participants did not provide demographic data). On average, AOM Time 1 participants were 42.01 years old (*SD* = 12.93 years). AOM Time 1 participants were also mostly White (*n* = 740; 80.7%), followed by race or ethnicities including African American (*n* = 51), Asian (*n* = 50), Hispanic (*n* = 27), Native American (*n* = 5), and other (*n* = 5). A total of 39 participants also identified as more than one racial or ethnic background. On average, participants reported having slightly less than a 4-year college degree (*M* = 3.68, *SD* = 0.85 on a 5-point education scale). All procedures were approved by University of Oregon Institutional Review Board.

### Procedure

Participants entered the online survey interface and consented to the study. Upon receiving informed consent, we solicited a range of responses, some of which were used in the current paper under our preregistration plan: risk perceptions associated with COVID-19, participants’ thoughts and feelings about the virus via a free response question, and dehumanization via the AOM. The free response question was the penultimate question, and the AOM question was the final question in the survey.

We also evaluated demographic items from a baseline wave (Wave 1; see Figure 2), including participant age, gender, and political ideology. Political ideology was assessed on a four-point scale (1 = very conservative, 4 = very liberal). Antecedents were therefore not assessed contemporaneously with the AOM measures.

### Preregistered Measures

Measures were identical for AOM Times 1 and 2 unless otherwise stated.

#### Blatant Dehumanization

We measured blatant dehumanization using the AOM scale (Kteily et al., 2015). Consistent with Markowitz and Slovic (2020b), participants rated the evolved nature of three groups on a scale of (1) Unevolved, to (8) Fully evolved: Asians, Asian Americans, and Americans. A fourth group, Chinese people, was added in AOM Time 2.

Dehumanization ratings were analyzed in two ways: (1) as absolute scores (e.g., low scores suggest more dehumanization on the AOM), and (2) as relative scores. Both measurements helped to evaluate if blatant dehumanization is connected to risk perceptions as an outright measure (e.g., people have decided and unqualified views of an entire outgroup during the pandemic) or a comparative measure (e.g., people have conditional or relative views of an entire outgroup during the pandemic compared to an ingroup). Relative dehumanization scores were calculated using the formula, [Americans – Target Group]. Here, high relative scores suggest people dehumanized an outgroup more than Americans (Kteily et al., 2015). The AOM scale was used in this paper because it is one of the more well-validated and recent measures of blatant outgroup dehumanization. According to Kteily et al. (2015), the AOM is a measure with high predictive validity relative to other measures of dehumanization and measures of prejudice. It is also linked to other known measures of blatant dehumanization (specifically, animalistic and mechanistic dehumanization), which adds to its convergent validity as well. We therefore adapted this measure to our study evaluating the dehumanization of Asian populations during the COVID-19 pandemic.

#### Risk Perceptions

Since COVID-19 was a new and unprecedented world event at the time of this project, we drew on established risk perception research and associated measures to assess how people perceived the COVID-19 pandemic in terms of risk (Brewer et al., 2007; Kahan et al., 2012; Ferrer et al., 2018). We substituted COVID-19 for other health risks (e.g., cancer, gum disease) using items from measures described by Ferrer et al. (2018) and adapted other items from prior work as well (Brewer et al., 2007; Kahan et al., 2012). Note, Ferrer et al. (2018) also describe how test-retest reliabilities for their measures were stable weeks after initial measurement, suggesting that our effects should also share a similar quality.

The first question of the risk perception index asked: “How likely is it that you will get the coronavirus at some point in the future?” on a scale of (1) Completely impossible to (6) Completely certain. The second question asked: “I think my chances of getting harmed by the coronavirus are:” on a scale of (1) Completely impossible to (6) Completely certain. The third question asked: “I think the consequences of the coronavirus for infected people are:” on a scale of (1) Not bad at all to (6) Extremely bad. The fourth question asked: “How easy or hard is it to imagine yourself getting infected with the coronavirus?” on a scale of (1) Extremely easy to (6) Extremely hard. The fifth question asked: “How fearful are you of contracting the coronavirus in the future?” on a scale of (1) Not fearful at all to (6) Extremely fearful. The sixth question asked: “Overall, how much risk do you believe the coronavirus poses to human health, safety, or prosperity in the United States?” on a scale of (1) No risk to (11) Extreme risk. Finally, the seventh question asked: “Compared to the average American, my chances of being infected with the coronavirus are:” on a scale of (1) Far below average to (6) Far above average.

Responses were averaged after reverse-scoring the fourth question and standardizing all items to form a *risk perception index* (AOM Time 1 Cronbach’s α = 0.82, AOM Time 2 Cronbach’s α = 0.83 using standardized items). The fourth question was reverse-scored for the index, only. High scores on this index indicate more perceived risk. Analyses using the index were also explored with each individual item for completeness and to assess the relative strength of each dimension. To support the convergent validity of our risk perception measure, we related the risk perception index to other antecedents that prior literature predicts should be theoretically associated. The data suggest males had lower risk perceptions at the index level than females in our sample [*t*(904) = 4.37, *p* < 0.001, Cohen’s *d* = 0.29], which is consistent with prior evidence (for a review, see Finucane et al., 2000). Test-retest reliabilities were highly significant across AOM timepoints for the risk perception index (*r* = 0.814, *p* < 0.001).

### Automated Text Analysis

All participants in AOM Time 1 responded to the following prompt: “We understand that coronavirus affects you, your community, and the world. We want to know what you are thinking and feeling about the coronavirus. Please be as detailed as possible.” Participants typed their responses in an untimed manner.

Responses were analyzed with Linguistic Inquiry and Word Count (LIWC: Pennebaker et al., 2015). LIWC is a text analysis tool for valid social and psychological evaluations of language (Tausczik and Pennebaker, 2010; Boyd and Pennebaker, 2015) and has been applied to evaluations of dehumanization as well (Markowitz and Slovic, 2020b). The tool contains an internal dictionary of words in social (e.g., family words), psychological (e.g., emotion terms), and part of speech categories (e.g., pronouns), and counts words as a percent of the total word count per text. We were interested in three categories *a priori*: negative affect, impersonal pronouns, and verbal drives.

#### Negative Affect

People who dehumanize tend to write with more negative affect (e.g., *hate*, *awful*) than people who do not dehumanize when describing an outgroup (Markowitz and Slovic, 2020b). In our study, people wrote about an event (e.g., COVID-19) potentially associated with outgroups (e.g., Asians, Asian Americans). We therefore assessed levels of negativity in participant writing to evaluate how dehumanization related to negative affect (H_1_).

#### Impersonal Pronouns

The rate of impersonal pronouns (e.g., *it*, *anyone*) considers how often people refer to objects or entities in an impersonal and distanced manner (Kacewicz et al., 2014; Markowitz and Slovic, 2020a). Often, when people use high rates of impersonal pronouns, they make indirect references to people, which can be considered an indicator of dehumanization (H_2_) (Markowitz and Slovic, 2020b).

#### Verbal Drives

To assess one’s implicit motives as reflected through writing style (H_3_), we measured the rate of verbal drives. Words in this category (e.g., *confident*, *ambition*, *control, overcome*) describe the degree to which people were self-determined.

### Exploratory Measures

#### Objective Numeracy (Wave 1)

Participants responded to four items that evaluated their numeric ability and received one point for each correct response. These items were adapted from prior numeracy measures (Cokely et al., 2012; Weller et al., 2013), and only four items were used to keep the participant effort burden reasonable in a long survey. Scores were combined into an objective numeracy index by adding the number of correct answers (min = 0, max = 4; Cronbach’s α = 0.54). The exact objective numeracy questions are located in the online supplement out of space considerations.

To support the convergent validity of our objective numeracy measure, we related the objective numeracy index to other antecedents that prior literature predicts should be associated. Males had higher objective numeracy scores than females [*t*(905) = 7.20, *p* < 0.001, Cohen’s *d* = 0.48], which is supported by prior work (Peters and Bjälkebring, 2015). More highly educated people also had greater objective numeracy (*r* = 0.177, *p* < 0.001), which is supported by prior work (Rolison et al., 2020).

#### Conspiracy and Disinformation Beliefs

We drew on prior reporting of COVID-19 conspiracy beliefs to measure participant agreement with three China-related conspiracies and disinformation (e.g., Lewis, 2020). These beliefs were measured on 6-point scales from (1) Strongly agree to (6) Strongly disagree. The first question stated, “Every new disease comes from China,” the second question stated, “It’s unsafe to go to Chinese restaurants right now,” and the third question stated, “The coronavirus is a biochemical weapon that leaked” (AOM Time 1 Cronbach’s α = 0.61, AOM Time 2 Cronbach’s α = 0.57 using standardized items). Inter-item correlations for these beliefs were significant and operated in the predicted direction for AOM Time 1 (average *r* = 0.340, all *p*s < 0.001) and AOM Time 2 (average *r* = 0.304, all *p*s < 0.001). Test-retest reliabilities were highly significant across AOM timepoints (average *r* = 0.656, *p*s < 0.001).

Descriptive statistics for all measures collapsed across participants are located in Table 1.

**TABLE 1:** Descriptive statistics for key variables in AOM Time 1 and AOM Time 2.

**AOM Time 1**	**Measure**	***M***	***SD***	**Q1**	***Mdn***	**Q3**
	AOM: Americans	7.25	1.29	7.00	8.00	8.00
	AOM: Asians	7.25	1.22	7.00	8.00	8.00
	AOM: Asian Americans	7.31	1.15	7.00	8.00	8.00
	AOM: Asians (relative)	0.00	1.01	0.00	0.00	0.00
	AOM: Asian Americans (relative)	-0.06	0.85	0.00	0.00	0.00
	Conspiracy belief: Every new disease comes from China	5.14	1.15	5.00	6.00	6.00
	Conspiracy belief: Unsafe to go to Chinese restaurant	4.49	1.52	4.00	5.00	6.00
	Conspiracy belief: COVID-19 biochemical weapon	4.83	1.37	4.00	5.00	6.00
	Negative affect (%)	4.27	3.14	2.13	3.87	5.88
	Impersonal pronouns (%)	7.03	3.80	4.43	6.67	9.30
	Verbal drives (%)	9.30	4.82	6.25	8.76	11.67
	Objective Numeracy Scale	1.86	1.05	1.00	2.00	2.00
	Risk perception index	0.00	0.70	-0.45	0.04	0.47
	Likely to get the virus	3.55	1.03	3.00	4.00	4.00
	Chances of being harmed by the virus	3.32	1.01	3.00	3.00	4.00
	Consequences of the virus	4.02	1.05	3.00	4.00	5.00
	Difficulty imagining the self-contracting the virus	2.87	1.28	2.00	3.00	4.00
	Fear of contracting the virus	3.48	1.52	2.00	3.00	5.00
	Degree to which the virus poses a health risk	8.50	2.13	7.50	9.00	10.00
	Chances of being infected with the virus	3.06	1.06	2.00	3.00	4.00
AOM Time 2	AOM: Americans	7.22	1.35	7.00	8.00	8.00
	AOM: Asians	7.19	1.29	7.00	8.00	8.00
	AOM: Asian Americans	7.29	1.16	7.00	8.00	8.00
	AOM: Chinese people	7.00	1.50	6.00	8.00	8.00
	AOM: Asians (relative)	0.03	1.10	0.00	0.00	0.00
	AOM: Asian Americans (relative)	-0.07	0.99	0.00	0.00	0.00
	AOM: Chinese people (relative)	0.22	1.31	0.00	0.00	0.00
	Conspiracy belief: Every new disease comes from China	5.01	1.24	5.00	5.00	6.00
	Conspiracy belief: Unsafe to go to Chinese restaurant	4.57	1.51	4.00	5.00	6.00
	Conspiracy belief: COVID-19 biochemical weapon	4.68	1.42	4.00	5.00	6.00
	Risk perception index	0.00	0.70	-0.48	0.03	0.52
	Likely to get the virus	3.36	1.05	3.00	3.00	4.00
	Chances of being harmed by the virus	3.13	1.02	2.00	3.00	4.00
	Consequences of the virus	3.93	1.16	3.00	4.00	5.00
	Difficulty imagining the self-contracting the virus	3.04	1.35	2.00	3.00	4.00
	Fear of contracting the virus	3.21	1.54	2.00	3.00	4.00
	Degree to which the virus poses a health risk	8.23	2.45	7.00	9.00	10.00
	Chances of being infected with the virus	2.91	1.07	2.00	3.00	4.00

*AOM = Ascent of Man. Labels with “(relative)” are dehumanization scores for the target group relative to Americans, where higher scores reflect more dehumanization toward the out-group relative to Americans. The risk perception index at each time point was a standardized composite variable.*

## Results

We used simple correlations to evaluate bivariate relationships between variables; Supplementary Table 2 contains associations after controlling for age and gender in multiple regression models. In general, the results were largely maintained in the regressions.

### AOM Time 1

#### Rates of Dehumanization

Using absolute dehumanization scores, we observed that 39.0% of our sample dehumanized Asians and 37.6% dehumanized Asian Americans (e.g., these individuals rated Asians or Asian Americans as less than fully human on the AOM). Compared to Americans (the relative scores), 10.4% of participants dehumanized Asians, and 6.1% dehumanized Asian Americans (e.g., these individuals rated Asians or Asian Americans as less human than Americans on the AOM).

#### Antecedents of Dehumanization: Political Ideology

Using absolute scores, Conservatives dehumanized Asians more than Liberals (*r* = 0.087, *p* = 0.008); no significant association emerged between conservativism and the dehumanization of Asian Americans (*r* = 0.034, *p* = 0.300). Using relative scores, Conservatives dehumanized both Asians (*r* = −0.301, *p* < 0.001) and Asian Americans (*r* = −0.277, *p* < 0.001) more than Liberals. These data are further described in Table 2. Note, for the creation of this table, political ideology was dichotomized from a four-point scale (1 = Very conservative, 2 = Conservative, 3 = Liberal, 4 = Very liberal) to a two-point scale (1 = Conservative, 2 = Liberal) for ease of interpretation and to be consistent with recent research on dehumanization (Markowitz and Slovic, 2020b). We also report the results of simple regressions between political ideology (continuous) and dehumanization in the online supplement (Supplementary Table 3), which revealed largely consistent findings.

**TABLE 2:** Dehumanization effects across political ideology and outgroups.

**AOM Time 1**	**Liberal (*n* = 546)**		**Conservative (*n* = 368)**				
	***M***	***SD***	***M***	***SD***	***t*(*df*)**	***p***	**Cohen’s *d***
Asians	7.31	1.13	7.15	1.35	-1.90 (689.79)	0.058	0.13
Asian Americans	7.32	1.10	7.29	1.22	-0.37 (912)	0.709	0.03
Asians (relative)	-0.21	0.99	0.31	0.95	7.90 (912)	<0.001	0.54
Asian Americans (relative)	-0.22	0.89	0.17	0.73	7.16 (875.58)	<0.001	0.47

**AOM Time 2**	**Liberal (*n* = 418)**		**Conservative (*n* = 302)**				
	***M***	***SD***	***M***	***SD***	***t* (*df*)**	***p***	**Cohen’s *d***

Asians	7.28	1.18	7.04	1.42	-2.39 (572.74)	0.017	0.18
Asian Americans	7.34	1.10	7.22	1.24	-1.42 (718)	0.156	0.11
Chinese people	7.18	1.30	6.74	1.72	-3.71 (535.47)	<0.001	0.29
Asians (relative)	-0.24	1.00	0.40	1.13	7.90 (601.71)	<0.001	0.60
Asian Americans (relative)	-0.29	0.99	0.23	0.91	7.27 (718)	<0.001	0.55
Chinese people (relative)	-0.13	1.02	0.71	1.50	8.41 (494.25)	<0.001	0.65

*AOM = Ascent of Man. Labels with “(relative)” are dehumanization scores for the target group relative to Americans, where higher scores reflect more dehumanization toward the out-group relative to Americans. Political ideology was dichotomized from our four-point scale for this table. Some participants did not provide a political ideology rating.*

#### Antecedents of Dehumanization: Gender and Age

Males and females had statistically similar rates of dehumanization toward Asians [*t*(905) = −1.82, *p* = 0.070, Cohen’s *d* = 0.12] and Asian Americans [*t*(905) = −1.71, *p* = 0.087, Cohen’s *d* = 0.11] and were therefore not significant at the 5% level. Using relative scores, females dehumanized Asian Americans more than males [Welch’s *t*(792.64) = −1.99, *p* = 0.047, Cohen’s *d* = 0.13], but not Asians (*p* = 0.156). Age was unrelated to dehumanization using both scoring methods (*p*s > 0.378).

#### Consequences of Dehumanization: Risk Perceptions

Using absolute scores, blatant dehumanization of Asian and Asian Americans was not significantly related to the risk perception index or any particular item (*p*s > 0.319). Using relative scores, the overall risk perception index was not associated with dehumanization of Asians at the 5% significance level (*r* = −0.063, *p* = 0.058; Table 3). Below, we explored this relationship at the item level to understand if all measures were indeed not significantly related to dehumanization.

**TABLE 3:** Bivariate correlations for AOM Time 1 (Top Panel) and AOM Time 2 (Bottom Panel) variables.

**AOM Time 1: Correlations with the Ascent of Man**							
**Category**	**Measure**	**Asians**	**Asian Americans**	**Asians (relative)**	**Asian Americans (relative)**	
Antecedents	Age	-0.006	-0.009	0.022	0.029	
	Political ideology	**0**.087**	0.034	**−0**.301**	**−0**.277**	
Consequences	Risk perception index	0.019	-0.004	-0.063	-0.042	
	Likely to get the virus	0.026	0.007	**−0**.065*	-0.049	
	Chances of being harmed by the virus	-0.005	-0.021	-0.024	-0.008	
	Consequences of the virus	0.004	-0.013	**−0**.085*	**−0**.077*	
	Difficulty imagining the self-contracting the virus	-0.022	0.001	**0**.078*	0.059	
	Fear of contracting the virus	-0.019	-0.018	0.005	0.003	
	Degree to which the virus poses a health risk	0.029	0.009	**−0**.080*	**−0**.065*	
	Chances of being infected with the virus	0.033	0.016	0.021	0.051	
Emotion	Negative affect	0.026	0.018	**−0**.070*	**−0**.069*	
Attention	Impersonal pronouns	0.030	0.034	0.001	-0.030	
Implicit motivation	Verbal drives	-0.046	-0.055	-0.011	-0.005	
Numeracy	Objective numeracy scale	0.016	0.017	**−0**.075*	**−0**.088**	
Conspiracy beliefs	Every new disease comes from China	**0**.254**	**0**.199**	**−0**.270**	**−0**.222**	
	Unsafe to go to Chinese restaurant	**0**.168**	**0**.131**	**−0**.190**	**−0**.161**	
	COVID-19 is a biochemical weapon	**0**.189**	**0**.174**	**−0**.199**	**−0**.198**	

**AOM Time 2: Correlations with the Ascent of Man**							
**Category**	**Measure**	**Asians**	**Asian Americans**	**Chinese people**	**Asians (relative)**	**Asian Americans (relative)**	**Chinese people (relative)**

Antecedents	Age	-0.017	-0.025	0.006	0.013	0.021	-0.013
	Political Ideology	**0**.112**	0.061	**0**.152**	**−0**.322**	**−0**.283**	**−0**.335**
Consequences	Risk perception index	**0**.073*	0.061	**0**.129**	**−0**.126**	**−0**.115**	**−0**.182**
	Likely to get the virus	0.030	0.021	0.041	-0.065	-0.058	-0.072
	Chances of being harmed by the virus	0.018	0.006	**0**.074*	-0.063	-0.054	**−0**.120**
	Consequences of the virus	0.064	0.046	**0**.138**	**−0**.086*	-0.067	**−0**.169**
	Difficulty imagining the self-contracting the virus	**−0**.105**	**−0**.093*	**−0**.117**	**0**.128**	0.115**	**0**.139**
	Fear of contracting the virus	-0.006	0.004	0.030	-0.066	−0.086*	**−0**.097**
	Degree to which the virus poses a health risk	0.072	0.062	**0**.126**	**−0**.133**	−0.126**	**−0**.185**
	Chances of being infected with the virus	0.072	0.063	**0**.104**	-0.072	-0.060	**−0**.110**
Numeracy	Objective numeracy scale	0.030	0.014	0.021	-0.049	-0.032	-0.036
Conspiracy beliefs	Every new disease comes from China	**0**.208**	**0**.153**	**0**.256**	**−0**.297**	**−0**.238**	**−0**.340**
	Unsafe to go to Chinese restaurant	**0**.167**	**0**.171**	**0**.149**	**−0**.184**	−0.188**	**−0**.161**
	COVID-19 is a biochemical weapon	**0**.188**	**0**.165**	**0**.227**	**−0**.272**	−0.251**	**−0**.305**

AOM = Ascent of Man. Labels with “(relative)” are dehumanization scores for the target group relative to Americans, where higher scores reflect more dehumanization toward the out-group relative to Americans. **p < 0.01, *p < 0.05. Significant relationships are bolded.

At the item level of the risk perceptions index, participants dehumanized Asians more than Americans if they believed they were less likely to get the coronavirus in the future (*r* = −0.065, *p* = 0.050), if they believed the consequences of the coronavirus were less severe for infected people (*r* = −0.085, *p* = 0.010), if it was difficult to imagine getting infected with the coronavirus (*r* = 0.078, *p* = 0.019), and if they believed the virus poses less of a human health risk (*r* = −0.080, *p* = 0.015). Dehumanization did not relate to risk beliefs about one’s perceived chances of being harmed by the virus, fear of contracting the virus, or perceived chances of being infected (see Table 3). People who dehumanized Asian Americans more than Americans believed the consequences of the coronavirus were less severe (*r* = −0.077, *p* = 0.020) and believed the virus poses less of a health risk (*r* = −0.065, *p* = 0.050). Such effects were non-significant after controlling for age, gender, and political ideology in the same model.

Inconsistent with H_1_, but consistent with the other risk perception results, higher rates of negative affect in participant writing were associated with less dehumanization of Asians (*r* = −0.070, *p* = 0.035) and Asian Americans (*r* = −0.069, *p* = 0.037) relative to Americans.

#### Consequences of Dehumanization: Impersonal Pronouns and Verbal Drives

Inconsistent with H_2_-_3_, rates of impersonal pronouns and verbal drives were statistically unrelated to dehumanization of Asians or Asian Americans for absolute and relative scores (*p*s > 0.096). Drives were positively associated with overall risk perceptions, however (*r* = 0.083, *p* = 0.012). See Supplementary Table 4 for bivariate correlations between all LIWC dimensions and dehumanization.

### AOM Time 1 Exploratory Findings

#### Objective Numeracy

Participants answered four math questions to indicate their numerical competency. Participants with lower objective numeracy scores indicated greater relative dehumanization of Asians (*r* = −0.075, *p* = 0.024) and Asian Americans (*r* = −0.088, *p* = 0.007) relative to Americans. Absolute dehumanization scores were unrelated to objective numeracy.

#### Conspiracy Beliefs

Considering both absolute and relative scores, people who dehumanized Asians and Asian Americans also agreed more with each of the three conspiracy theories (Asians: *r*s > |0.168|, *p* < 0.001; Asian Americans: *r*s > |0.131|, *p* < 0.001). Considering both scores, the conspiracy theory most strongly associated with dehumanization was the idea that every new disease comes from China (*r*s > | 0.199|, *p* < 0.001). Finally, less numerate people tended to agree more with all three conspiracy beliefs in AOM Time 1 (*r*s > 0.079, *p*s < 0.017).

To further investigate this novel finding, we regressed dehumanization scores on objective numeracy and reported level of education, which can serve as a rough proxy for intelligence, in general. Using relative scores, numeracy predicted dehumanization of Asians in the expected direction (*B* = −0.08, *SE* = 0.03, *t* = −2.38, *p* = 0.018, *R*^2^ = 0.006), but education was not related to dehumanization (*B* = 0.03, *SE* = 0.04, *t* = 0.86, *p* = 0.389). For Asian Americans, numeracy predicted dehumanization (*B* = −0.07, *SE* = 0.03, *t* = −2.59, *p* = 0.010, *R*^2^ = 0.008) and education did not (*B* = −0.01, *SE* = 0.03, *t* = −0.33, *p* = 0.743). Together, one’s proficiency with numbers is likely powering the prior effects, not one’s general intelligence or level of education. Note, since absolute dehumanization scores were unrelated to objective numeracy, we did not investigate this relationship controlling for education.

### AOM Time 2

Using the same participants from AOM Time 1, we attempted to replicate our findings and evaluate how changes over time might have affected dehumanization ratings. AOM Time 2 was conducted more than 1 month after AOM Time 1. We instituted two changes in AOM Time 2, however: (1) the inclusion of Chinese people to the AOM scale, and (2) we did not proceed with the automated text analyses since the preregistered effects largely failed to obtain significance. All AOM Time 2 bivariate correlations are reported in the bottom of Table 3.

#### Rates of Dehumanization

Slightly elevated levels of dehumanization occurred in AOM Time 2 versus AOM Time 1: 41.2% of our sample dehumanized Asians (compared to 39.0% in AOM Time 1), 38.9% dehumanized Asian Americans (37.6% in AOM Time 1), and 44.4% dehumanized Chinese people using absolute scores. Compared to Americans, 12.2% (10.4% in AOM Time 1) and 7.6% (6.1% in AOM Time 1) of participants dehumanized Asians and Asian Americans, respectively. A greater proportion, 17.7%, dehumanized Chinese people using relative scores.

The increase in AOM dehumanization over time toward Asians (using absolute scores) was confirmed by a significant paired samples *t*-test, though effects failed to obtain for other outgroups and scoring calculations (Table 4).

**TABLE 4:** Changes in AOM dehumanization across timepoints.

	**AOM Time 1**		**AOM Time 2**				
**Perceived outgroup**	***M***	***SD***	***M***	***SD***	***t*(722)**	***p***	**Cohen’s *d***
Asians	7.26	1.22	7.19	1.29	2.23	0.026	0.08
Asian Americans	7.33	1.14	7.29	1.16	1.21	0.227	0.05
Asians (relative)	-0.001	1.00	0.03	1.10	-1.16	0.246	0.04
Asian Americans (relative)	-0.07	0.85	-0.07	0.99	0.05	0.960	0.002

*Results are from paired samples *t*-tests. Labels with “(relative)” are dehumanization scores for the target group relative to Americans. For absolute scores, lower ratings suggest more dehumanization. For relative scores, higher ratings suggest more dehumanization of the outgroup relative to Americans.*

#### Antecedents of Dehumanization: Political Ideology

Conservatives dehumanized Asians and Chinese people more than Liberals using both absolute and relative scores (*r*s>|0.112|, *p*s < 0.003). Conservatives dehumanized Asian Americans more than Liberals using relative scores, only (*r* = −0.283, *p* < 0.001).

**FIGURE 3:**
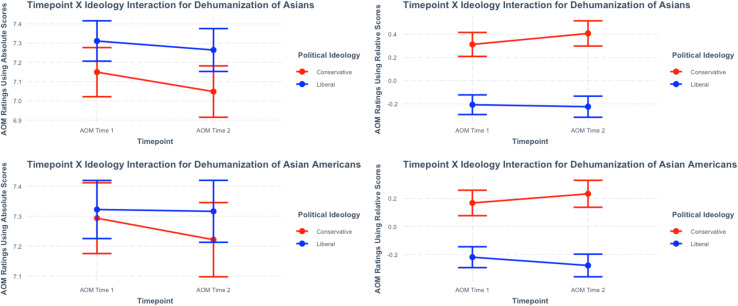
Timepoint (AOM Time 1, AOM Time 2) × Political Ideology (Liberal, Conservative) interaction effects. Error bars are 95% Confidence Intervals. For absolute scores, lower ratings suggest more dehumanization. For relative scores, higher ratings suggest more dehumanization.

Who dehumanized more over time? Given that political ideology is typically one of the strongest antecedents connected to dehumanization (Bruneau and Kteily, 2017; Utych, 2018; Markowitz and Slovic, 2020b), we used ideology to address this question directly. Consistent with other work (Markowitz and Slovic, 2020b), we dichotomized our political ideology variable and performed Timepoint (AOM Time 1, AOM Time 2) × Political Ideology (Liberal, Conservative) interactions (Figure 3). A significant interaction emerged for Asians using relative scores [*F*(1, 763.04) = 3.82, *p* = 0.051], with Conservatives displaying an increase in dehumanization from AOM Time 1 (*M* = 0.31, *SE* = 0.05) to AOM Time 2 (*M* = 0.41, *SE* = 0.06), *p* = 0.030. Liberals did not show a significant change from AOM Time 1 to 2 (*p* = 0.643).

The Timepoint (AOM Time 1, AOM Time 2) × Political Ideology (Liberal, Conservative) interaction was also significant for Asian Americans using relative scores [*F*(1, 774.62) = 5.27, *p* = 0.022], with only a trend toward Conservatives dehumanizing Asian Americans more than Americans from AOM Time 1 (*M* = 0.17, *SE* = 0.05) to AOM Time 2 (*M* = 0.23, *SE* = 0.05), *p* = 0.115. Liberals tended to reveal a decrease in dehumanization toward Asian Americans from AOM Time 1 (*M* = −0.22, *SE* = 0.04) to AOM Time 2 (*M* = −0.28, *SE* = 0.04), *p* = 0.092, but not at the 5% significance level. No interaction effects emerged for absolute scores (*p*s > 0.290). Note, these reported interaction effects were largely maintained if the continuous political ideology measure was used for Asians relative to Americans (*p* = 0.040) and Asian Americans relative to Americans (*p* = 0.073) (see Supplementary Figure 1).

#### Antecedents of Dehumanization: Gender and Age

Males dehumanized Chinese people more than females [Welch’s *t*(676.33) = −3.01, *p* = 0.003, Cohen’s *d* = 0.22], but no other associations between gender and dehumanization were significant. Age was unrelated to dehumanization across scoring methods in AOM Time 2 (*p*s > 0.497).

#### Consequences of Dehumanization: Risk Perceptions

More dehumanization of Asians was associated with lower risk perceptions using absolute scores (*r* = 0.073, *p* = 0.050). Using relative scores, more dehumanization of Asians was associated with reduced perceived severity of the virus and its consequences (*r* = −0.086, *p* = 0.020), increased difficulty imagining the self-contracting the virus (*r* = 0.128, *p* = 0.001), and reduced beliefs that the virus poses a human health risk (*r* = −0.133, *p* < 0.01).

The absolute score of dehumanization of Asian Americans was not significantly associated with the risk perception index (*r* = 0.061, *p* = 0.103). Consistent with AOM Time 1, however, dehumanization of Asian Americans relative to Americans was associated with reduced beliefs that the virus posed a human health risk (*r* = −0.126, *p* = 0.01).

Dehumanization of Chinese people (relative to Americans) was associated with reduced risk perceptions at the index level (*r* = −0.182, *p* < 0.001). Specifically, people who dehumanized Chinese people more than Americans tended to believe that their chances of being harmed by the virus were lower (*r* = −0.120, *p* = 0.001) and the consequences of the virus were less severe (*r* = −0.169, *p* < 0.001). They also indicated greater difficulty in imagining the self-contracting the virus (*r* = 0.139, *p* < 0.001), less fear of contracting the virus (*r* = −0.097, *p* = 0.009), lower perceived risk of the virus to human health (*r* = −0.185, *p* < 0.001), and beliefs that their chances of being infected with the virus was less than the average American (*r* = −0.110, *p* = 0.003).

### AOM Time 2 Exploratory Findings

#### Objective Numeracy

Less numerate people tended to have greater agreement with all conspiracy beliefs in AOM Time 2 (*r*s > 0.118, *p* < 0.001), except for the belief that it is unsafe to patronize Chinese restaurants (*r* = 0.030, *p* = 0.414).

#### Conspiracy Beliefs

Across all analytic approaches and target groups (i.e., Asians, Asian Americans, Chinese people), more dehumanization was associated with greater agreement with each of the three conspiracy beliefs (see bottom panel of Table 3).

### Within-Subjects Changes

To evaluate how participant ratings might have changed across timepoints, we calculated difference scores between each variable using the following formula: [AOM Time 2 – AOM Time 1]. Higher scores on each measure suggest an increase on a particular dimension over time from AOM Time 1 to AOM Time 2. At the bivariate level (Supplementary Table 5), there were no significant changes across timepoints except for the conspiracy belief that every new disease comes from China. The results suggest that an increase in this conspiracy belief over time was associated with more dehumanization toward Asians and Asian Americans over time as well, but only for absolute scores.

To analyze these data in a different way, we explored the connection between dehumanization and one of our key variables, the risk perceptions index, using linear mixed models including a fixed effect for Timepoint (AOM Time 1, AOM Time 2) and a random intercept for participant. The results are presented in the online supplement for transparency (Supplementary Table 6) and suggest greater dehumanization toward Asians and Asian Americans (relative to Americans) was associated with reduced risk perceptions after accounting for Timepoint and multiple observations from the same participant. This more sophisticated analysis might pick up additional important within-subject variation that simple change scores fail to adequately account for.

## Discussion

In a two-wave study, we predicted that dehumanization would be associated with greater perceived risk and negative affect toward COVID-19. Our results indicated the opposite pattern with more dehumanization connected to perceiving the virus as *less* of a problem. People who dehumanized Asians and Asian Americans relative to Americans tended to believe the consequences of the virus were less severe, posed less of a health risk, and they believed in harmful disinformation about the virus. People who dehumanized also wrote with less negative affect when providing their thoughts and feeling about the pandemic. We observed that the dehumanization of Chinese people (AOM Time 2) was more prevalent than other outgroups, on average. These results are empirically reasonable in the sense that COVID-19 originated in China and people might ascribe more (undeserved) blame toward people from this location.

Many statistical effects in this paper emerged using relative scores but not absolute scores. Why did these patterns emerge? Relative scores might be more appropriate to capture less-than-human perceptions because some people might not have a clear, unconditional opinion of an outgroup. Instead, their dehumanization might only appear when the outgroup is directly compared to the ingroup (Haslam, 2014). It is possible that people in our study did not believe Asians, Asian Americans, or Chinese people were less than fully human during the pandemic, but relative to Americans, they were comparatively viewed as “less than.”

Several contributions of this research are worth highlighting. First, we measured perceptions of outgroups who are connected to, but unjustly blamed for, a pandemic as the global crisis unfolded. These perceptions were recruited in a two-wave study design that offers a substantial advancement to the dehumanization literature. On average, Conservatives but not Liberals dehumanized Asians relative to Americans more over time (Figure 3). Therefore, just as the virus and recommendations to combat its effects changed, feelings toward specific outgroups appeared to change as well. This is possibly due to certain media types communicating anti-Asian ideas during the pandemic (for theoretical support, see Kasperson et al., 1988). The tendency to dehumanize is therefore situationally influenced, subject to change, and ideologically polarized. Communicators may be able to address dehumanization and reduce it (i.e., discrediting conspiracies; showing potential outgroups as part of “us”). However, such messages should be carefully designed and tested in target groups given the potential for reactance (Dillard and Shen, 2005). Feeding a dehumanizing rhetoric (e.g., calling COVID-19 the “China Virus”) promotes division and undermines an ethnic group’s humanity when society needs unity and decency instead (Somvichian-Clausen, 2020).

Second, this work observed dehumanization is associated with less objective numeracy and more conspiracy beliefs about the virus (see Table 3). To the best of our knowledge, this is the first study to observe that people who are objectively better at numerical problems and operations tend to perceive outgroups as more human. Plausible explanations for this pattern include: (1) those higher on objective numeracy are often more logical decision makers than those lower on objective numeracy, and (2) numeracy is not the only indicator of dehumanization. Note, we included a measure of verbal intelligence in a post-hoc analysis, though due to low psychometric properties, it was omitted. Future work should continue to explore if other measures and forms of intelligence can reliably predict dehumanization as well.

People who dehumanized tended to believe the virus was less risky, which might suggest they would be less willing to heed the advice of public health officials (e.g., wearing masks, maintaining social distance), inevitably increasing their own risk and the risk of others for infection. Why did these individuals dehumanize? One possibility is that dehumanization might be a form of motivated perception. People who dehumanize might perceive target outgroups to be psychologically and physically distant “others.” As a result of this distanced view, dehumanizers might perceive themselves as less susceptible to disease. Alternatively, these data might represent people who dehumanize abstracting away from COVID-19 in some manner; believing that its consequences are less severe, and they cannot imagine the self being infected. Recall, prior work also suggests that people may dehumanize others to cope with social and psychological stress. For example, doctors may dehumanize patients to manage the demands of their profession and distance themselves from care burden (Bursztajn et al., 1990; Schulman-Green, 2003; Haque and Waytz, 2012). Although dehumanizing medical patients is clearly unequal to dehumanizing an entire ethnic group, people who dehumanize during the COVID-19 pandemic might use a similar psychological coping strategy to manage distress across settings. Dehumanization may help to reappraise and reconceptualize a target group, though prior work suggests this might be a maladaptive coping strategy (see Martin and Dahlen, 2005; Garnefski and Kraaij, 2006).

Unjustly alienating, harming, and perceiving an outgroup as less than human weakens virus recovery efforts that require all people be treated fairly, decently, and humanely. We hope that by identifying how dehumanization is associated with thoughts, feelings, and risk perceptions about the virus, we can mitigate such harmful treatment now and in the future. Developing tools or interventions to prevent dehumanization, perhaps by increasing interpersonal contact between ingroup and outgroup members (Capozza et al., 2017), might be a fruitful line of research to explore in future work.

### Limitations and Future Directions

Our study used a convenience sample of American participants and therefore, we only have dehumanization perceptions from one country. It would be important to evaluate the widespread nature of dehumanization of Asian populations, and especially Chinese populations, across the world. Second, our effect sizes ranged from small to medium. Our ability to detect these effects benefitted from our large-scale survey and a post-hoc power analysis indeed confirmed that we achieved approximately 86% power estimating small effects in AOM Wave 1 (*r* = 0.10, α = 0.05, two-tailed). Future research should continue to collect large samples to ensure adequately powered science. Third, our evidence is correlational and both causal directions are plausible: (1) dehumanization led to decreased risk perceptions, or (2) decreased risk perceptions led to dehumanization. Experimental evidence should complement our current study. Future work should use this evidence of dehumanization and develop potential interventions for those who exhibit warning signs of treating others as “less than.” It might also be illustrative to investigate if the effects reported in this paper are amplified or attenuated after isolating by specific participant demographics (e.g., race, gender; Finucane et al., 2000). Finally, we omitted some measures (e.g., verbal intelligence) due to their low psychometric properties. Future work should consider how to best measure and evaluate other intelligence measures in their connection in dehumanization.

## Conclusion

The evidence in this two-wave study suggests a nontrivial number of Americans dehumanized Asians, Asian Americans, and Chinese people during early stages of the COVID-19 pandemic. These individuals tended to be ideologically conservative and believe the virus was less of a problem during late March 2020 and May 2020. Dehumanization remains an important psychological construct to evaluate during the pandemic and how people treat purported outgroups can be predicted by their risk perceptions of the virus.

## Data Availability Statement

The raw data supporting the conclusions of this article will be made available by the authors, without undue reservation.

## Ethics Statement

The studies involving human participants were reviewed and approved by Research Compliance Services, University of Oregon. Written informed consent for participation was not required for this study in accordance with the national legislation and the institutional requirements.

## Author Contributions

DM wrote the manuscript and analyzed the data. All authors provided critical feedback and edits on the manuscript.

## Conflict of Interest

The authors declare that the research was conducted in the absence of any commercial or financial relationships that could be construed as a potential conflict of interest.

## Footnotes

1 https://osf.io/xzthg/
