# Supplementary material for: Dehumanization During the COVID-19 Pandemic
Source: Front Psychol. 2021 Feb 11;12:634543. doi: 10.3389/fpsyg.2021.634543 (PMC7904886; doi:10.3389/fpsyg.2021.634543)
Supplement: Supplementary file 1 [file Data_Sheet_1.docx]

**Supplementary Materials: Table of Contents**

[Correlation Matrix for Risk Perception Items 2](#_Toc62021928)

[Multiple Regression Results Controlling for Participant Age and Gender 3](#_Toc62021929)

[Dehumanization Effects Treating Political Ideology as a Continuous Variable 6](#_Toc62021930)

[Bivariate Correlations Between AOM Ratings and Language Dimensions in AOM Time 1 7](#_Toc62021931)

[Supplementary Figure S1: Timepoint X Ideology Interaction Effects 10](#_Toc62021932)

[Bivariate Relationships Between Change Scores from AOM Time 1 to AOM Time 2 11](#_Toc62021933)

[Linear Mixed Effects Models Predicting Dehumanization from Risk Perceptions and Time 12](#_Toc62021934)

[Objective Numeracy Questions (Cronbach’s α = 0.54) 13](#_Toc62021935)

[Correlation Matrix of Objective Numeracy Questions 13](#_Toc62021936)

Supplementary Table S1

# Correlation Matrix for Risk Perception Items

| AOM Time 1 | | | | | | | |
| --- | --- | --- | --- | --- | --- | --- | --- |
| Item # | Risk perception item | 1 | 2 | 3 | 4 | 5 | 6 |
| 1 | Likely to get the virus | -- |  |  |  |  |  |
| 2 | Chances of being harmed by the virus | .710^**^ | -- |  |  |  |  |
| 3 | Consequences of the virus | .207^**^ | .316^**^ | -- |  |  |  |
| 4 | Difficulty imagining the self contracting the virus | -.495^**^ | -.422^**^ | -.146^**^ | -- |  |  |
| 5 | Fear of contracting the virus | .440^**^ | .541^**^ | .446^**^ | -.311^**^ | -- |  |
| 6 | Degree to which the virus poses a health risk | .313^**^ | .374^**^ | .555^**^ | -.232^**^ | .508^**^ | -- |
| 7 | Chances of being infected with the virus | .593^**^ | .556^**^ | .204^**^ | -.359^**^ | .431^**^ | .250^**^ |
| AOM Time 2 | | | | | | | |
| Item # | Risk perception item | 1 | 2 | 3 | 4 | 5 | 6 |
| 1 | Likely to get the virus | -- |  |  |  |  |  |
| 2 | Chances of being harmed by the virus | .687^**^ | -- |  |  |  |  |
| 3 | Consequences of the virus | .151^**^ | .330^**^ | -- |  |  |  |
| 4 | Difficulty imagining the self contracting the virus | -.491^**^ | -.406^**^ | -.189^**^ | -- |  |  |
| 5 | Fear of contracting the virus | .414^**^ | .555^**^ | .492^**^ | -.320^**^ | -- |  |
| 6 | Degree to which the virus poses a health risk | .258^**^ | .394^**^ | .604^**^ | -.252^**^ | .533^**^ | -- |
| 7 | Chances of being infected with the virus | .588^**^ | .551^**^ | .205^**^ | -.389^**^ | .442^**^ | .252^**^ |

*Note*. ***p* < .01. The fourth item of each scale was reverse-scored for the creation of the risk perception index, but its original coding is retain in this table.

Supplementary Table S2

# Multiple Regression Results Controlling for Participant Age and Gender

|  |  | AOM Time 1 | | | |  | AOM Time 2 | | | |
| --- | --- | --- | --- | --- | --- | --- | --- | --- | --- | --- |
| Dehumanization Independent Variable | Dependent Variable | *B* | *SE* | *t* | *p* |  | *B* | *SE* | *t* | *p* |
| Asians | Risk perception index | 0.01 | 0.02 | 0.35 | .725 |  | 0.04 | 0.02 | 1.80 | .072 |
|  | Likely to get the virus | 0.02 | 0.03 | 0.62 | .534 |  | 0.02 | 0.03 | 0.65 | .519 |
|  | Chances of being harmed by the virus | -0.01 | 0.03 | -0.32 | .747 |  | 0.01 | 0.03 | 0.33 | .743 |
|  | Consequences of the virus | 0.01 | 0.03 | 0.21 | .836 |  | 0.06 | 0.03 | 1.79 | .074 |
|  | Difficulty imagining the self contracting the virus | -0.02 | 0.03 | -0.55 | .582 |  | -0.11 | 0.04 | -2.86 | .004 |
|  | Fear of contracting the virus | -0.04 | 0.04 | -1.02 | .310 |  | -0.02 | 0.04 | -0.52 | .604 |
|  | Degree to which the virus poses a health risk | 0.05 | 0.06 | 0.82 | .414 |  | 0.13 | 0.07 | 1.82 | .069 |
|  | Chances of being infected with the virus | 0.02 | 0.03 | 0.73 | .464 |  | 0.06 | 0.03 | 1.79 | .074 |
|  | Political ideology | **0.06** | **0.02** | **2.62** | **.009** |  | **0.07** | **0.02** | **2.97** | **.003** |
|  | Objective numeracy | 0.03 | 0.03 | 1.04 | .299 |  | 0.04 | 0.03 | 1.30 | .193 |
|  | Negative affect | 0.07 | 0.09 | 0.79 | .428 |  | 0.06 | 0.09 | 0.70 | .483 |
|  | Conspiracy belief: Every new disease comes from China | **0.25** | **0.03** | **8.05** | **< .001** |  | **0.20** | **0.04** | **5.78** | **< .001** |
|  | Conspiracy belief: Unsafe to go to Chinese restaurant | **0.21** | **0.04** | **5.17** | **< .001** |  | **0.19** | **0.04** | **4.41** | **< .001** |
|  | Conspiracy belief: COVID-19 biochemical weapon | **0.22** | **0.04** | **6.10** | **< .001** |  | **0.22** | **0.04** | **5.33** | **< .001** |
|  |  |  |  |  |  |  |  |  |  |  |
| Asian Americans | Risk perception index | -0.01 | 0.02 | -0.28 | .783 |  | 0.03 | 0.02 | 1.49 | .138 |
|  | Likely to get the virus | 0.00 | 0.03 | 0.07 | .948 |  | 0.01 | 0.03 | 0.40 | .688 |
|  | Chances of being harmed by the virus | -0.02 | 0.03 | -0.77 | .442 |  | 0.00 | 0.03 | 0.05 | .957 |
|  | Consequences of the virus | -0.01 | 0.03 | -0.32 | .751 |  | 0.05 | 0.04 | 1.25 | .212 |
|  | Difficulty imagining the self contracting the virus | 0.00 | 0.04 | 0.05 | .961 |  | **-0.11** | **0.04** | **-2.59** | **.010** |
|  | Fear of contracting the virus | -0.04 | 0.04 | -0.88 | .381 |  | -0.01 | 0.05 | -0.20 | .841 |
|  | Degree to which the virus poses a health risk | 0.01 | 0.06 | 0.20 | .842 |  | 0.12 | 0.08 | 1.56 | .119 |
|  | Chances of being infected with the virus | 0.01 | 0.03 | 0.29 | .770 |  | 0.05 | 0.03 | 1.53 | .126 |
|  | Political ideology | 0.02 | 0.02 | 1.07 | .285 |  | 0.04 | 0.03 | 1.52 | .130 |
|  | Objective numeracy | 0.03 | 0.03 | 0.99 | .321 |  | 0.03 | 0.03 | 0.81 | .418 |
|  | Negative affect | 0.05 | 0.09 | 0.52 | .600 |  | 0.08 | 0.10 | 0.79 | .431 |
|  | Conspiracy belief: Every new disease comes from China | **0.20** | **0.03** | **6.22** | **< .001** |  | **0.17** | **0.04** | **4.20** | **< .001** |
|  | Conspiracy belief: Unsafe to go to Chinese restaurant | **0.18** | **0.04** | **4.06** | **< .001** |  | **0.22** | **0.05** | **4.59** | **< .001** |
|  | Conspiracy belief: COVID-19 biochemical weapon | **0.22** | **0.04** | **5.64** | **< .001** |  | **0.22** | **0.05** | **4.69** | **< .001** |
|  |  |  |  |  |  |  |  |  |  |  |
| Asians relative to Americans | Risk perception index | **-0.05** | **0.02** | **-2.28** | **.023** |  | **-0.08** | **0.02** | **-3.49** | **.001** |
|  | Likely to get the virus | **-0.08** | **0.03** | **-2.43** | **.015** |  | **-0.07** | **0.04** | **-2.01** | **.045** |
|  | Chances of being harmed by the virus | **-0.03** | **0.03** | **-1.01** | **.311** |  | -0.07 | 0.03 | -1.93 | .054 |
|  | Consequences of the virus | **-0.09** | **0.04** | **-2.44** | **.015** |  | **-0.08** | **0.04** | **-2.12** | **.035** |
|  | Difficulty imagining the self contracting the virus | **0.11** | **0.04** | **2.50** | **.013** |  | **0.16** | **0.05** | **3.37** | **.001** |
|  | Fear of contracting the virus | -0.02 | 0.05 | -0.40 | .688 |  | **-0.10** | **0.05** | **-1.97** | **.049** |
|  | Degree to which the virus poses a health risk | **-0.21** | **0.07** | **-2.88** | **.004** |  | **-0.29** | **0.08** | **-3.48** | **.001** |
|  | Chances of being infected with the virus | 0.02 | 0.04 | 0.59 | .556 |  | **-0.08** | **0.04** | **-2.13** | **.034** |
|  | Political ideology | **-0.24** | **0.03** | **-9.26** | **< .001** |  | **-0.23** | **0.03** | **-8.91** | **< .001** |
|  | Objective numeracy | **-0.07** | **0.03** | **-1.96** | **.050** |  | -0.04 | 0.04 | -1.11 | .267 |
|  | Negative affect | **-0.24** | **0.11** | **-2.26** | **.024** |  | -0.17 | 0.11 | -1.60 | .111 |
|  | Conspiracy belief: Every new disease comes from China | **-0.33** | **0.04** | **-8.75** | **< .001** |  | **-0.34** | **0.04** | **-8.31** | **< .001** |
|  | Conspiracy belief: Unsafe to go to Chinese restaurant | **-0.31** | **0.05** | **-6.13** | **< .001** |  | **-0.27** | **0.05** | **-5.34** | **< .001** |
|  | Conspiracy belief: COVID-19 biochemical weapon | **-0.28** | **0.05** | **-6.08** | **< .001** |  | **-0.35** | **0.05** | **-7.43** | **< .001** |
|  |  |  |  |  |  |  |  |  |  |  |
| Asian Americans relative to Americans | Risk perception index | -0.05 | 0.03 | -1.81 | .070 |  | **-0.09** | **0.03** | **-3.25** | **.001** |
|  | Likely to get the virus | **-0.09** | **0.04** | **-2.06** | **.039** |  | -0.07 | 0.04 | -1.86 | .064 |
|  | Chances of being harmed by the virus | -0.03 | 0.04 | -0.61 | .540 |  | -0.07 | 0.04 | -1.78 | .076 |
|  | Consequences of the virus | **-0.09** | **0.04** | **-2.16** | **.031** |  | -0.07 | 0.04 | -1.47 | .141 |
|  | Difficulty imagining the self contracting the virus | **0.11** | **0.05** | **2.10** | **.036** |  | **0.16** | **0.05** | **3.04** | **.002** |
|  | Fear of contracting the virus | -0.05 | 0.06 | -0.77 | .441 |  | **-0.15** | **0.06** | **-2.64** | **.008** |
|  | Degree to which the virus poses a health risk | **-0.22** | **0.09** | **-2.50** | **.013** |  | **-0.31** | **0.09** | **-3.31** | **.001** |
|  | Chances of being infected with the virus | 0.06 | 0.04 | 1.40 | .163 |  | -0.07 | 0.04 | -1.82 | .070 |
|  | Political ideology | **-0.27** | **0.03** | **-8.53** | **< .001** |  | **-0.22** | **0.03** | **-7.59** | **< .001** |
|  | Objective numeracy | **-0.09** | **0.04** | **-2.20** | **.028** |  | -0.02 | 0.04 | -0.48 | .632 |
|  | Negative affect | **-0.29** | **0.13** | **-2.26** | **.024** |  | -0.21 | 0.12 | -1.78 | .075 |
|  | Conspiracy belief: Every new disease comes from China | **-0.33** | **0.05** | **-7.19** | **< .001** |  | **-0.30** | **0.05** | **-6.48** | **< .001** |
|  | Conspiracy belief: Unsafe to go to Chinese restaurant | **-0.33** | **0.06** | **-5.31** | **< .001** |  | **-0.31** | **0.06** | **-5.55** | **< .001** |
|  | Conspiracy belief: COVID-19 biochemical weapon | **-0.34** | **0.06** | **-6.12** | **< .001** |  | **-0.35** | **0.05** | **-6.73** | **< .001** |
|  |  |  |  |  |  |  |  |  |  |  |
| Chinese people | Risk perception index | -- | -- | -- | -- |  | **0.05** | **0.02** | **3.05** | **.002** |
|  | Likely to get the virus | -- | -- | -- | -- |  | 0.03 | 0.03 | 1.06 | .288 |
|  | Chances of being harmed by the virus | -- | -- | -- | -- |  | 0.04 | 0.03 | 1.43 | .152 |
|  | Consequences of the virus | -- | -- | -- | -- |  | **0.11** | **0.03** | **3.64** | **< .001** |
|  | Difficulty imagining the self contracting the virus | -- | -- | -- | -- |  | **-0.11** | **0.03** | **-3.24** | **.001** |
|  | Fear of contracting the virus | -- | -- | -- | -- |  | 0.00 | 0.04 | 0.01 | .990 |
|  | Degree to which the virus poses a health risk | -- | -- | -- | -- |  | **0.18** | **0.06** | **2.86** | **.004** |
|  | Chances of being infected with the virus | -- | -- | -- | -- |  | **0.07** | **0.03** | **2.57** | **.010** |
|  | Political ideology | -- | -- | -- | -- |  | **0.08** | **0.02** | **4.02** | **< .001** |
|  | Objective numeracy | -- | -- | -- | -- |  | 0.04 | 0.03 | 1.56 | .119 |
|  | Negative affect | -- | -- | -- | -- |  | -- | -- | -- | -- |
|  | Conspiracy belief: Every new disease comes from China | -- | -- | -- | -- |  | **0.23** | **0.03** | **7.51** | **< .001** |
|  | Conspiracy belief: Unsafe to go to Chinese restaurant | -- | -- | -- | -- |  | **0.15** | **0.04** | **3.87** | **< .001** |
|  | Conspiracy belief: COVID-19 biochemical weapon | -- | -- | -- | -- |  | **0.23** | **0.04** | **6.60** | **< .001** |
|  |  |  |  |  |  |  |  |  |  |  |
| Chinese people relative to Americans | Risk perception index | -- | -- | -- | -- |  | **-0.10** | **0.02** | **-4.72** | **< .001** |
|  | Likely to get the virus | -- | -- | -- | -- |  | **-0.07** | **0.03** | **-2.30** | **.022** |
|  | Chances of being harmed by the virus | -- | -- | -- | -- |  | **-0.09** | **0.03** | **-2.99** | **.003** |
|  | Consequences of the virus | -- | -- | -- | -- |  | **-0.14** | **0.03** | **-4.23** | **< .001** |
|  | Difficulty imagining the self contracting the virus | -- | -- | -- | -- |  | **0.15** | **0.04** | **3.75** | **< .001** |
|  | Fear of contracting the virus | -- | -- | -- | -- |  | **-0.10** | **0.04** | **-2.22** | **.027** |
|  | Degree to which the virus poses a health risk | -- | -- | -- | -- |  | **-0.32** | **0.07** | **-4.46** | **< .001** |
|  | Chances of being infected with the virus | -- | -- | -- | -- |  | **-0.09** | **0.03** | **-2.99** | **.003** |
|  | Political ideology | -- | -- | -- | -- |  | **-0.20** | **0.02** | **-9.37** | **< .001** |
|  | Objective numeracy | -- | -- | -- | -- |  | -0.04 | 0.03 | -1.44 | .150 |
|  | Negative affect | -- | -- | -- | -- |  | -- | -- | -- | -- |
|  | Conspiracy belief: Every new disease comes from China | -- | -- | -- | -- |  | **-0.35** | **0.03** | **-10.10** | **< .001** |
|  | Conspiracy belief: Unsafe to go to Chinese restaurant | -- | -- | -- | -- |  | **-0.20** | **0.04** | **-4.58** | **< .001** |
|  | Conspiracy belief: COVID-19 biochemical weapon | -- | -- | -- | -- |  | **-0.34** | **0.04** | **-8.68** | **< .001** |

*Note*. Dashes suggest a variable was not measured during a particular timepoint. Significant associations (*p* < .05) are bolded.

Supplementary Table S3

# Dehumanization Effects Treating Political Ideology as a Continuous Variable

| AOM Time 1 | *B* | *SE* | *t* | *p* | *R^2^* |
| --- | --- | --- | --- | --- | --- |
| Asians | 0.13 | 0.05 | 2.65 | .008 | 0.008 |
| Asian Americans | 0.05 | 0.05 | 1.04 | .300 | 0.001 |
| Asians (relative) | -0.38 | 0.04 | -9.54 | < .001 | 0.091 |
| Asian Americans (relative) | -0.30 | 0.03 | -8.72 | < .001 | 0.077 |
| AOM Time 2 | *B* | *SE* | *t* | *p* | *R^2^* |
| Asians | 0.18 | 0.06 | 3.03 | .003 | 0.013 |
| Asian Americans | 0.09 | 0.05 | 1.63 | .103 | 0.004 |
| Chinese people | 0.29 | 0.07 | 4.12 | < .001 | 0.023 |
| Asians (relative) | -0.45 | 0.05 | -9.12 | < .001 | 0.104 |
| Asian Americans (relative) | -0.35 | 0.05 | -7.90 | < .001 | 0.080 |
| Chinese people (relative) | -0.55 | 0.06 | -9.52 | < .001 | 0.112 |

*Note*. These results were calculated using simple regression models.

Supplementary Table S4

# Bivariate Correlations Between AOM Ratings and Language Dimensions in AOM Time 1

|  | Ratings on the AOM Connected to Language Variables | | | |
| --- | --- | --- | --- | --- |
|  | Asians | Asian Americans | Asians  (relative to Americans) | Asian Americans (relative to Americans) |
| WC | .058 | .049 | −.070^*^ | −.066^*^ |
| Analytic | −.095^**^ | −.091^**^ | .004 | −.009 |
| Clout | .005 | .003 | −.001 | .002 |
| Authentic | .025 | .030 | .042 | .045 |
| Tone | .001 | −.013 | .012 | .033 |
| WPS | −.055 | −.059 | .004 | .005 |
| Sixltr | .012 | −.006 | −.075^*^ | −.065 |
| Dic | .057 | .070^*^ | .055 | .052 |
| function | .010 | .025 | .057 | .048 |
| pronoun | .055 | .050 | .004 | .017 |
| ppron | .039 | .029 | .004 | .022 |
| i | .031 | .026 | .018 | .030 |
| we | −.006 | −.006 | .003 | .004 |
| you | −.036 | −.043 | .056 | .073^*^ |
| shehe | .031 | .011 | −.084^*^ | −.070^*^ |
| they | .038 | .041 | −.024 | −.030 |
| ipron | .030 | .034 | .001 | −.002 |
| article | −.049 | −.031 | .009 | −.018 |
| prep | −.071^*^ | −.057 | .037 | .018 |
| auxverb | .100^**^ | .101^**^ | −.019 | −.015 |
| adverb | .086^**^ | .096^**^ | .010 | .006 |
| conj | −.049 | −.039 | .042 | .033 |
| negate | −.012 | −.030 | −.050 | −.037 |
| verb | .067^*^ | .062 | −.004 | .007 |
| adj | .009 | .020 | −.017 | −.035 |
| compare | −.021 | −.016 | −.025 | −.037 |
| interrog | .061 | .052 | −.027 | −.015 |
| number | −.013 | −.020 | −.018 | −.013 |
| quant | .032 | .047 | .021 | .006 |
| affect | .047 | .030 | −.069^*^ | −.054 |
| posemo | .019 | .001 | −.027 | −.007 |
| negemo | .026 | .018 | −.070^*^ | −.069^*^ |
| anx | .057 | .057 | −.007 | −.003 |
| anger | −.006 | −.018 | −.070^*^ | −.066^*^ |
| sad | −.018 | −.003 | .018 | .000 |
| social | .021 | .002 | −.023 | .001 |
| family | .000 | −.010 | .044 | .066^*^ |
| friend | −.014 | −.008 | .024 | .019 |
| female | .035 | .022 | −.002 | .018 |
| male | .023 | −.007 | −.078^*^ | −.051 |
| cogproc | .022 | .012 | −.015 | −.001 |
| insight | −.001 | −.013 | .008 | .025 |
| cause | .031 | .020 | −.080^*^ | −.077^*^ |
| discrep | −.003 | .005 | .037 | .032 |
| tentat | .028 | .015 | −.003 | .016 |
| certain | .014 | .028 | .040 | .030 |
| differ | −.024 | −.035 | −.016 | −.005 |
| percept | −.043 | −.035 | .021 | .011 |
| see | −.006 | .014 | .025 | .003 |
| hear | −.038 | −.035 | −.007 | −.016 |
| feel | −.016 | −.018 | .002 | .003 |
| bio | −.059 | −.048 | .093^**^ | .090^**^ |
| body | −.031 | −.046 | .009 | .027 |
| health | −.040 | −.025 | .089^**^ | .081^*^ |
| sexual | −.014 | −.012 | .007 | .005 |
| ingest | −.040 | −.036 | .039 | .037 |
| drives | −.046 | −.055 | −.011 | −.005 |
| affiliation | −.002 | −.006 | .037 | .049 |
| achieve | −.017 | −.032 | −.048 | −.039 |
| power | −.019 | −.035 | −.065^*^ | −.057 |
| reward | −.059 | −.063 | .053 | .061 |
| risk | −.015 | .002 | −.045 | −.077^*^ |
| focuspast | .008 | .004 | −.052 | −.057 |
| focuspresent | .054 | .054 | .001 | .005 |
| focusfuture | .020 | .007 | −.006 | .012 |
| relativ | .028 | .041 | .029 | .018 |
| motion | .003 | .009 | .002 | −.005 |
| space | −.002 | .000 | .024 | .025 |
| time | .046 | .068^*^ | .053 | .037 |
| work | −.015 | −.014 | −.044 | −.053 |
| leisure | −.009 | −.014 | −.026 | −.025 |
| home | −.012 | −.009 | .004 | .000 |
| money | .007 | .000 | −.028 | −.023 |
| relig | .006 | .010 | .035 | .038 |
| death | −.054 | −.081^*^ | .026 | .062 |
| informal | .005 | −.012 | −.073^*^ | −.063 |
| swear | −.012 | −.021 | −.084^*^ | −.088^**^ |
| netspeak | −.052 | −.070^*^ | −.019 | −.003 |
| assent | −.006 | −.012 | −.007 | −.001 |
| nonflu | .039 | .031 | −.007 | .006 |
| filler | .010 | .012 | −.016 | −.020 |
| AllPunc | .032 | .009 | −.111^**^ | −.097^**^ |
| Period | .021 | .011 | −.023 | −.011 |
| Comma | .003 | −.001 | −.051 | −.054 |
| Colon | −.065^*^ | −.025 | .054 | .005 |
| SemiC | .041 | .044 | −.002 | −.002 |
| QMark | −.042 | −.043 | .018 | .020 |
| Exclam | −.054 | −.048 | .014 | .004 |
| Dash | .024 | .010 | −.044 | −.033 |
| Quote | −.028 | −.041 | −.016 | −.005 |
| Apostro | .052 | .037 | −.107^**^ | −.102^**^ |
| Parenth | −.032 | −.055 | −.056 | −.037 |
| OtherP | .038 | .024 | −.030 | −.013 |

*Note*. ***p* < .01, * *p* < .05.


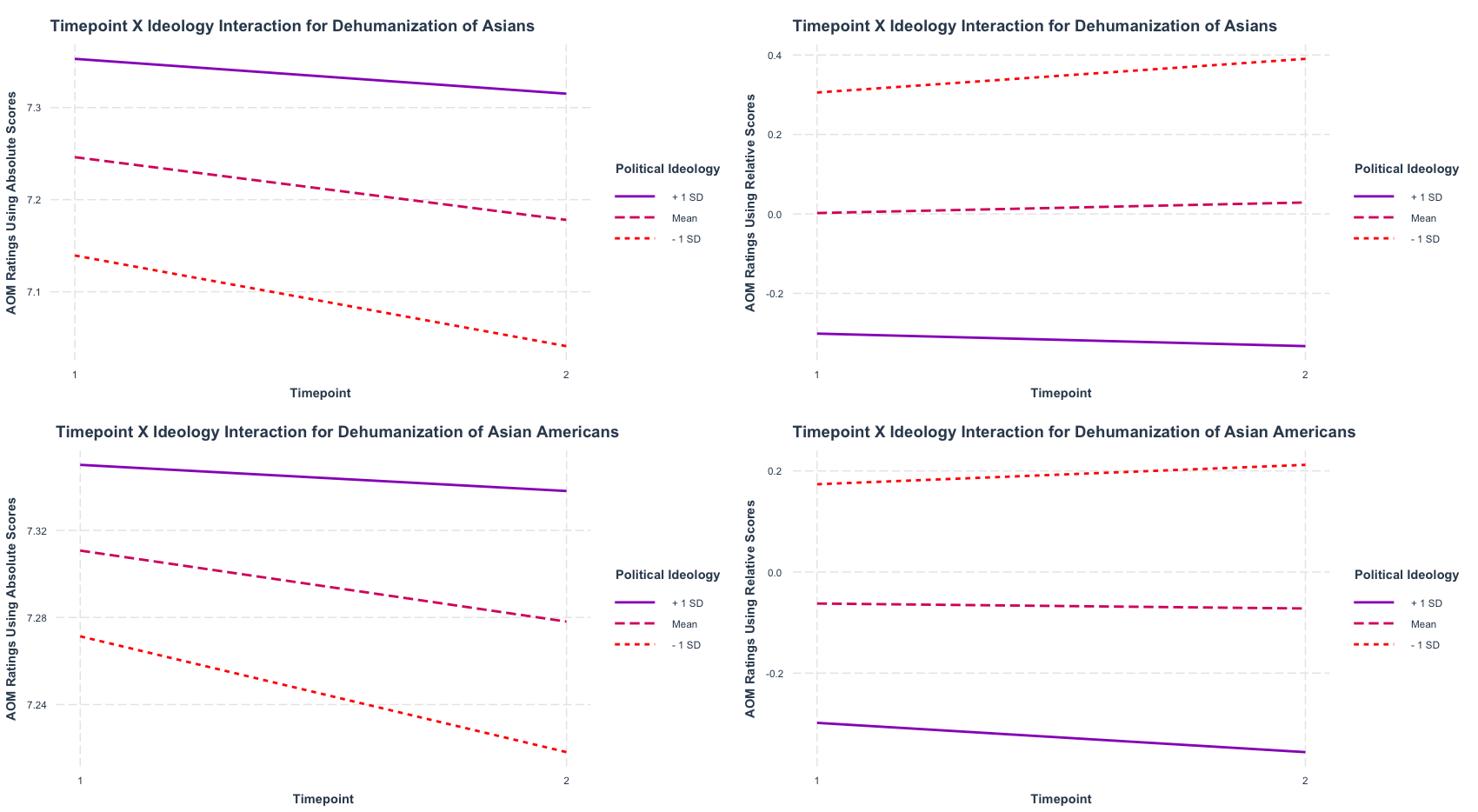
Supplementary Figure S1: Timepoint X Ideology Interaction Effects

*Note*. Timepoint 1 = AOM Time 1 and Timepoint 2 = AOM Time 2.

Supplementary Table S5

# Bivariate Relationships Between Change Scores from AOM Time 1 to AOM Time 2

| Category | Measure | Change: Asians | Change: Asian Americans | Change: Asians (relative) | Change: Asian Americans (relative) |
| --- | --- | --- | --- | --- | --- |
| Risk perceptions | Risk perception index | .020 | .008 | -.020 | -.007 |
|  | Likely to get the virus | -.017 | -.036 | .011 | .033 |
|  | Chances of being harmed by the virus | -.027 | -.029 | .022 | .024 |
|  | Consequences of the virus | .010 | -.013 | .035 | .064 |
|  | Difficulty imagining the self contracting the virus | -.056 | -.041 | .065 | .049 |
|  | Fear of contracting the virus | -.039 | -.027 | -.026 | -.042 |
|  | Degree to which the virus poses a health risk | .066 | .065 | -.030 | -.027 |
|  | Chances of being infected with the virus | .025 | .031 | -.022 | -.028 |
| Numeracy | Objective numeracy scale | .058 | .025 | .017 | .059 |
| Conspiracy beliefs | Every new disease comes from China | **.105^**^** | **.099^**^** | -.009 | .004 |
|  | Unsafe to go to Chinese restaurant | -.021 | -.016 | .041 | .036 |
|  | COVID-19 is a biochemical weapon | .036 | .021 | -.072 | -.057 |

*Note*. Labels with “(relative)” are dehumanization scores for the target group relative to Americans. Higher scores reflect more dehumanization toward the out-group relative to Americans. ***p* < .01, * *p* < .05. Change scores were used for each variable in this table (except for objective numeracy, which was assessed in the baseline Wave 1; see Figure 2 in the main text) and calculated using the following formula: AOM Time 2 – AOM Time 1. Significant relationships (*p* < .05) are bolded.

Supplementary Table S6

| Asians | | | | | |
| --- | --- | --- | --- | --- | --- |
| Fixed effects | *B* | *SE* | *t* | *p* | *R^2^c* |
| Risk perception index | 0.06 | 0.05 | 1.23 | .220 | 0.758 |
| AOM Time 2 (ref = AOM Time 1) | -0.07 | 0.03 | -2.22 | .027 |  |
| Random effects | σ^2^ | *SD* |  |  |  |
| Participant | 1.19 | 1.09 |  |  |  |
| Asian Americans | | | | | |
| Fixed effects | *B* | *SE* | *t* | *p* | *R^2^c* |
| Risk perception index | 0.02 | 0.04 | 0.54 | .587 | 0.739 |
| AOM Time 2 (ref = AOM Time 1) | -0.03 | 0.03 | -1.12 | .263 |  |
| Random effects | σ^2^ | *SD* |  |  |  |
| Participant | 0.99 | 0.99 |  |  |  |
| Asians (relative) | | | | | |
| Fixed effects | *B* | *SE* | *t* | *p* | *R^2^c* |
| Risk perception index | -0.10 | 0.04 | -2.57 | .010 | 0.728 |
| AOM Time 2 (ref = AOM Time 1) | 0.03 | 0.03 | 1.18 | .239 |  |
| Random effects | σ^2^ | *SD* |  |  |  |
| Participant | 0.79 | 0.89 |  |  |  |
| Asian Americans (relative) | | | | | |
| Fixed effects | *B* | *SE* | *t* | *p* | *R^2^c* |
| Risk perception index | -0.07 | 0.04 | -2.05 | .041 | 0.677 |
| AOM Time 2 (ref = AOM Time 1) | -0.01 | 0.03 | -0.15 | .883 |  |
| Random effects | σ^2^ | *SD* |  |  |  |
| Participant | 0.56 | 0.75 |  |  |  |

# Linear Mixed Effects Models Predicting Dehumanization from Risk Perceptions and Time

*Note*. Ref = reference group. *R^2^c* = variance explained by the fixed and random effects from the MuMIn package in *R*.

# Objective Numeracy Questions (Cronbach’s α = 0.54)

1. Suppose that you are buying a gallon of milk at the grocery store. There are two options for the same brand of milk: buying 4 quarts at $2.50 per quart or buying 1 gallon for $8.00. What is the cost per quart (1 gallon=4 quarts) of the better priced milk? $______ per quart
2. Imagine you are throwing a fair six-sided die (the sides of which show 1, 2, 3, 4, 5, 6) 120 times. On average, how many times would you expect this die to show a number less than 5 (1, 2, 3 or 4)? ______ out of 120 throws.
3. Out of 300 fruits, 200 are apples and 100 are bananas. Out of the 200 apples, 90 are green. Out of the 100 bananas, 30 are green. What is the probability that a randomly picked green fruit will be an apple? ____ %
4. In a field 40% of snakes are striped, 30% brown and 30% black. A striped snake is poisonous with a probability of 10%. A snake that is not striped is poisonous with a probability of 20%. What is the probability that a poisonous snake in the field is striped? __ %

# Correlation Matrix of Objective Numeracy Questions

|  | Q1 | Q2 | Q3 |
| --- | --- | --- | --- |
| Q1 | -- |  |  |
| Q2 | .183^**^ | -- |  |
| Q3 | .124^**^ | .348^**^ | -- |
| Q4 | .120^**^ | .199^**^ | .390^**^ |

*Note*. ** *p* < .01. These correlations are expectedly small because the items were developed using item-response theory, not classical testing theory, which favors inter-item correlations.
